# Fracture Resistance of Equine Cheek Teeth With and Without Occlusal Fissures: A Standardized *ex vivo* Model

**DOI:** 10.3389/fvets.2021.699940

**Published:** 2021-09-07

**Authors:** Elke Pollaris, Bart J. G. Broeckx, Sivaprakash Rajasekharan, Rita Cauwels, Lieven Vlaminck

**Affiliations:** ^1^Department of Surgery and Anaesthesiology of Domestic Animals, Faculty of Veterinary Medicine, Ghent University, Ghent, Belgium; ^2^Department of Nutrition, Genetics and Ethology, Faculty of Veterinary Medicine, Ghent University, Ghent, Belgium; ^3^Department of Paediatric Dentistry and Special Care, PAECOMEDIS Research Cluster, Ghent University, Ghent, Belgium

**Keywords:** equine, cheek tooth, fracture, fracture resistance, fissure

## Abstract

**Background:***Ex vivo* fracture models are frequently used in human dentistry to provide insights in the fracture mechanisms of teeth. Equine cheek teeth fractures are an important dental pathology, but there has been no research performed to examine the fracture resistance *ex vivo*.

**Objective:** To evaluate the fracture resistance of equine cheek teeth and identify anatomical predictors that might influence fracture resistance in healthy teeth. It was further evaluated if the presence of a fissure caused a decrease in fracture resistance.

**Study design:***Ex vivo* experimental design.

**Methods:** Individual cheek teeth were subjected to a compression load in a universal testing machine until fracture occurred. Testing was performed in two study groups. A first group of healthy cheek teeth was tested to examine anatomical predictors on fracture resistance. A second group comprised cheek teeth with occlusal fissures and an equal number of age- and size-matched fissure-free teeth as controls. The effect of possible predictors on fracture resistance was investigated by regression analysis.

**Results:** In the first group, fracture resistance was significantly influenced by the location on the tooth where testing was performed in both maxillary (*p* < 0.001) and mandibular teeth (*p* < 0.001). Additional significantly associated factors were Triadan number in mandibular (*p* = 0.009) and the mesiodistal length of the occlusal surface of maxillary teeth (*p* = 0.01). Experimentally induced crown fractures that extended below the simulated bone level were more frequently associated with pulp horn exposure (*p* < 0.001). In the second group, significant lower fracture loads were recorded in teeth with fissures (mandibular *p* = 0.006; maxillary *p* < 0.001), compared to fissure-free teeth.

**Main limitations:** This *ex vivo* model does not imitate the *in vivo* masticatory forces and lacks the shock-absorbing properties of the periodontal ligament.

**Conclusions:** The methodology used in this study provides an *ex vivo* experimental set-up to test fracture resistance of equine cheek teeth enabling evidence-based research to examine the potentially weakening effects of tooth pathology and its treatments. Crown resistance to fracture differed along the occlusal surface of healthy equine cheek teeth, and the presence of fissures further decreased fracture resistance.

## Introduction

The cheek teeth of horses play a very important role in the horse's digestive strategy since it is characterized by a high chewing efficiency (1). During the mastication process considerable forces are developed which have an effect on the tooth (and its surrounding structures) (2, 3). However, it is not known to which extent an equine tooth can cope with these forces and when the critical point to fracture is reached.

Equine cheek teeth fractures are considered an important dental pathological disorder which can have serious consequences for the well-being of the horse. Different fracture patterns have been described, but only the etiology of maxillary sagittal midline fractures has been discovered. This fracture type is considered to develop secondary to advanced infundibular caries and therefore is referred to as caries related infundibular fractures (4–6). However, other fracture patterns remain idiopathic (4–9). It has been suggested that these idiopathic fractures occur on sites of structural weakness (5), but the fracture resistance of equine cheek teeth and the possible difference in fracture tolerance at specific locations on the tooth has not been examined up to date. Furthermore, information is lacking whether there are characteristics of the tooth (e.g., age-dependent changes) that influence the ability to withstand masticatory forces. In human teeth it has been demonstrated that young teeth have a higher fracture susceptibility because of their wide root canals and relative lower presence of mineralized tissues (dentin) (10, 11). In contrast, the median age of horses with a fractured tooth is reported to be 11–12 years (6, 8), which indicates a decrease in strength with age. Recently, attention has been brought to the presence of fissures on the occlusal surface of equine cheek teeth and their ability to progress into gross crown fractures (12). Therefore, we hypothesize that the presence of occlusal fissures causes a lower fracture resistance compared to cheek teeth without fissures.

The aim of this study was to determine the fracture resistance of equine cheek teeth in an *ex vivo* experiment. Hereby, it was intended to examine the possible differences in fracture resistance between specific locations on the tooth, the effect of age and the effect of the dimensions of the occlusal surface. Additionally, the impact of the presence of a fissure on fracture resistance was examined.

## Materials and Methods

### Study Design and Study Populations

An *ex vivo* experimental study was conducted on individual cheek teeth to examine their fracture resistance. Equine cheek teeth (Triadan 07–10) were extracted post-mortem (within 24 h after dead) from horses either euthanized for non-dental related problems or obtained from an abattoir in Belgium in 2020. Clinical information was available for the euthanized horses and the age of cadavers from the abattoir was estimated by mandibular incisor examination by one person (EP) (13). Only teeth without any signs of clinical dental pathology (except fissures) were included in the study. Surrounding tissues (bone, periodontal ligament) were removed from the teeth and the occlusal surface was inspected for the absence or presence of fissures. When a fissure was present, the fissure type (Table 1) (14) and the location of the fissure on the occlusal surface were recorded. Macro photographic images (5MP, Samsung galaxy A51) were taken of the occlusal surface of all teeth to ensure that no fissures were missed. The width (buccal-lingual distance) and length (mesio-distal distance) of each tooth's occlusal surface was measured with a caliper. Teeth were stored in 0.5% chloramine T trihydrate at 4°C until further processing.

**Table 1:** Fissure classification (14).

**Fissure type**	**Definition**
Type 1	Fissures that involve the secondary dentin on the occlusal surface
1a	Fissure orientation is perpendicular to the surrounding enamel fold, variably involving the adjacent enamel or even the peripheral cementum
1b	Fissure orientation does not follow a perpendicular orientation in relation to one surrounding enamel fold. Often this orientation is more mesio-distal.
Type 2	Fissures that do not involve the secondary dentin

High-resolution X-ray computed tomography (μCT) imaging was performed on teeth with fissures at the in-house developed μCT system HECTOR (15) of the Ghent University Centre for X-ray Tomography (UGCT). Covering a rotation of 360°, 2,000 projection images were made at an exposure time of 1,000 ms each. Using geometrical magnification, an isotropic voxel size of 50.1363 μm3 is achieved in the reconstructed volume, reconstructed using Octopus Reconstruction. At the source parameters of 150 kV and 30 W target power, the influence of spot blurring is negligible. Beam hardening was countered both in hardware by adding 1 mm Al filter and in the reconstruction software. The depth of each fissure was measured as illustrated in Supplementary Image 1.

**Figure 1:**
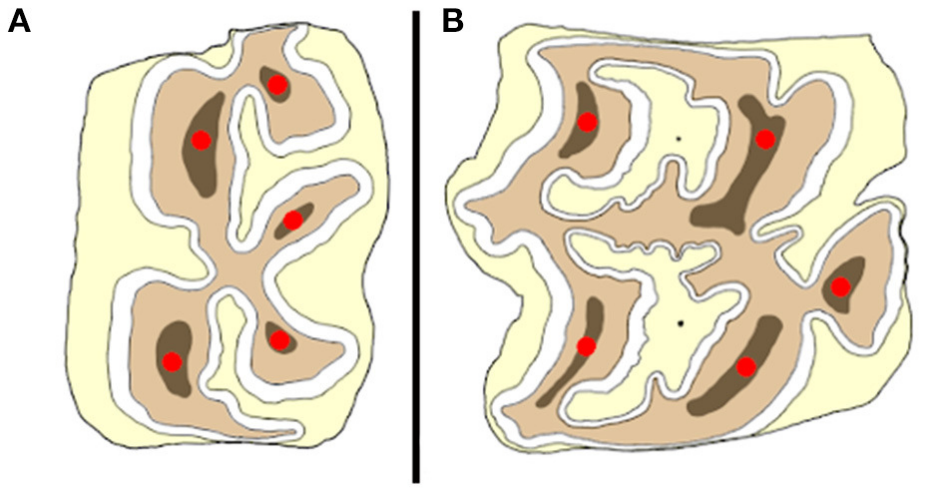
Schematic illustration of the placement of the tip of the compression device (red dots) on a mandibular **(A)** and maxillary **(B)** cheek tooth at different anatomical locations.

Testing was performed in two study groups. The number of teeth used was based on the availability of cadaver heads. The first study population consisted of healthy cheek teeth without abnormalities to examine individual and anatomical predictors that could influence the fracture resistance of the tooth. In this group only teeth of horses with a known age were used. A schematic representation of the different occlusal locations where mechanical pressure was exerted is illustrated in Figure 1. One or two locations were tested on the same tooth (a second location was tested only if the first loaded site did not result in a fracture involving a significant part of the tooth and after re-inspection of the tooth for induced cracks in the remaining part of the tooth). The second study population comprised cheek teeth with macroscopically identified occlusal fissures, and an equal number of age- and size-matched fissure-free teeth selected as controls. Mechanical testing of sites where a fissure type 1a was present, was performed by placing the tip of the device on the location where the fissure entered the secondary dentin. The same approach was performed for type 1b fissures. When a type 1b fissure involved the secondary dentine above two different pulp horns, the tip was placed on the secondary dentin at the side where the fissure ran closest to/ breached through the outer enamel ring. For type 2 fissures, the tip was positioned on the most axial site of the fissure on the occlusal surface. The tip was positioned on the same site for matched teeth.

### *Ex vivo* Fracture Resistance Set-Up

**Figure 2:**
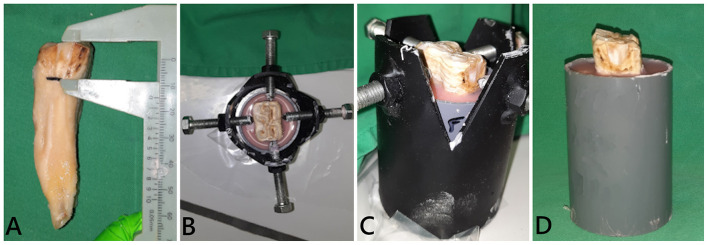
Embedding of a tooth in resin blocks. **(A)** The simulated bone level was determined on a tooth stripped of periodontal ligament. The tooth was fixed in a custom-made holder at the desired height and position [**(B)** dorsal view; **(C)** side view]. **(D)** Final tooth fixation.

The teeth were embedded in resin blocks (Palapress®, Kulzer Benelux, Haarlem, The Netherlands) within polyvinyl chloride (PVC) cylinders (5 cm outer diameter, 8–9 cm high). The level of the resin was fixed at 15 mm below the occlusal surface for every tooth, measured from the mid-point of the tooth at the interproximal surfaces, to approximate the normal bone level on the tooth (Figure 2). Immediately upon setting of the resin, the specimen was placed in cool water to dissipate the heat of polymerization of the resin. To prevent dehydration of the mounted tooth until final processing, the specimens were kept in demineralized water.

**Figure 3:**
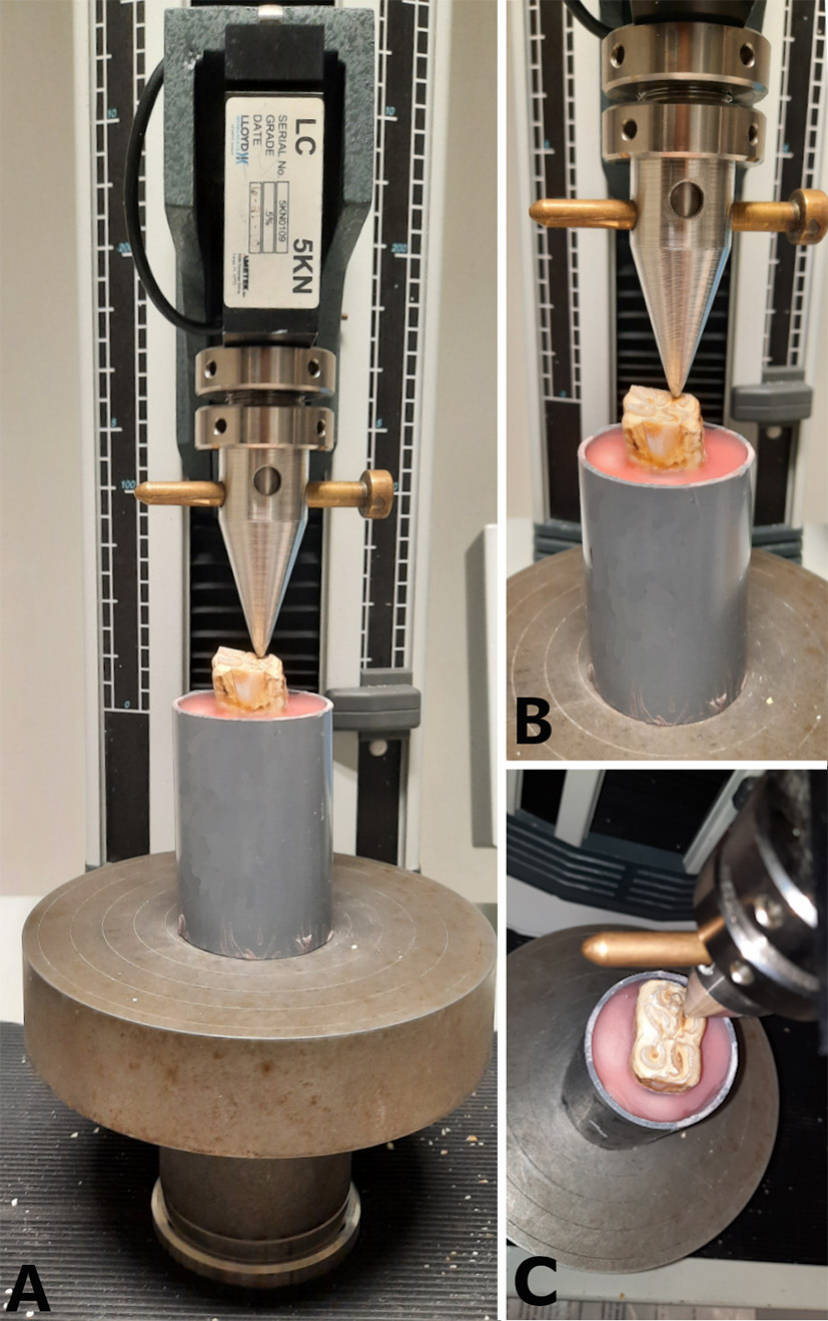
Representation of the application of a compressive force to the occlusal surface of a mandibular cheek tooth at the level of the secondary dentine above pulp horn 2. **(A)** Frontal view of the specimen placed under the compression device attached to a 5,000 N load cell. **(B)** Close up of the front view centered on the tooth. **(C)** Dorsal view of the positioning of the compression device.

Between 2 and 8 h after the embedding process, specimens were subjected to a load at a crosshead speed of 1 mm.min^−1^ in a universal testing machine (LRX plus, loadcell 5000N, LLOYD instruments, Ametek Inc.) until a fracture occurred. For the purpose of this study, point pressure was chosen to be able to test different areas on the occlusal surface. Pressure was exerted with a custom-made compression device (triangular shape, tip diameter 2 mm) which was positioned on the predefined site on the occlusal surface (Figure 3). The failure (fracture) load (N) of each site was recorded. The highest force prior to fracture was considered the maximum force sustained by the tooth. Inspection of the tooth after fracturing included recording of the fracture pattern, fracture level (above, equal to or below the simulated bone level), and whether the pulp cavity was exposed or not.

### Data Analysis

Categorical variables were expressed as numbers and percentages. Continuous variables were presented as mean ± standard deviation (and range, when interesting). Two major outcomes were assessed (fracture resistance and fracture level) in two different groups of teeth.

In the first study group (teeth with no abnormalities), the fracture resistance and the fracture level (above/equal/below the simulated bone level) of mandibular and maxillary cheek teeth were compared using mixed models with mandible/maxilla as fixed effect and horse as random effect. The effect of the fracture level (independent variable) on the occurrence of pulp exposure (dependent variable) was compared with a mixed model with horse as random effect. The age of the teeth (dependent variable) with and without pulp exposure (independent variable) was also compared with a mixed model with horse as random effect.

For the subsequent analyses, mandibular and maxillary cheek teeth were analyzed separately due to the anatomical difference between the respective teeth. An ANOVA was conducted to compare the fracture resistance between horses to assess whether there was variability by horse. Subsequently, effect of individual predictors, i.e., gender (male/female), jaw side (left/right), tooth number (Triadan 07 – 10), age of the tooth, tooth side (lingual/buccal), location on the tooth [secondary dentine above pulp horn (SD-PH) 1 – 5] and occlusal surface width, length and surface area (width × length) on fracture resistance was assessed with a linear mixed model with the individual predictor as fixed effect and horse as random effect. Furthermore, the effect of location on the tooth on fracture level was also assessed. Significance was assessed with a likelihood ratio test. *P*-values were corrected for multiple testing by multiplying them by the number of tests and are reported as such. Next, the most optimal generalized mixed model was determined based on the combination of predictors that minimized the Akaike Information.

In the second study group, the overall average fracture resistance of cheek teeth with and without fissures was compared with a linear mixed model with the presence of fissure (yes/no) as fixed effect and horse as random effect. The fracture level of teeth with and without fissures was also compared with a mixed model with horse as random effect. A similar approach as described for the first study group with separate analysis for mandibular and maxillary cheek teeth was used for the remainder of the analyses, i.e., a mixed model with the addition of presence of fissure (yes/no) and fissure type (0, 1a, 1b, 2) as individual predictors.

For all mixed model analyses with a categorial variable with more than 2 categories as the dependent variable, the Begg and Gray Approximation was used. The overall significance was set at α ≤ 0.05. The program R version 4.0.2 (“Taking off again”) was used for all analyses (16).

## Results

### *Ex vivo* Fracture Resistance of Equine Cheek Teeth With No Abnormalities

Fifty-nine healthy cheek teeth from seven horses (four mares, three geldings) were included to examine possible factors that influenced fracture resistance of equine cheek teeth. These included 28 mandibular cheek teeth with a mean dental age of 9.0 ± 2.0 years (min 5 y, max 12 y), 8 of which were Triadan 07s, 7 Triadan 08s, 7 Triadan 09s and 6 Triadan 10s. Their average occlusal surface width was 19.9 ± 1.5 mm (min 17 mm, max 23 mm) and the average occlusal surface length was 28.4 ± 1.5 mm (min 26 mm, max 31 mm). Furthermore, 31 maxillary cheek teeth with a mean dental age of 9.2 ± 2.0 years old (min 5 y, max 12 y) were included, consisting of 7 Triadan 07s, 10 Triadan 08s, 5 Triadan 09s and 9 Triadan 10s. These teeth had an average occlusal surface width of 28.9 ± 1.3 mm (min 27 mm, max 31 mm) and an average occlusal surface length of 28.1 ± 1.6 mm (min 25 mm, max 31 mm). An overview of the locations tested per tooth can be found in Supplementary Information 1.

**Figure 4:**
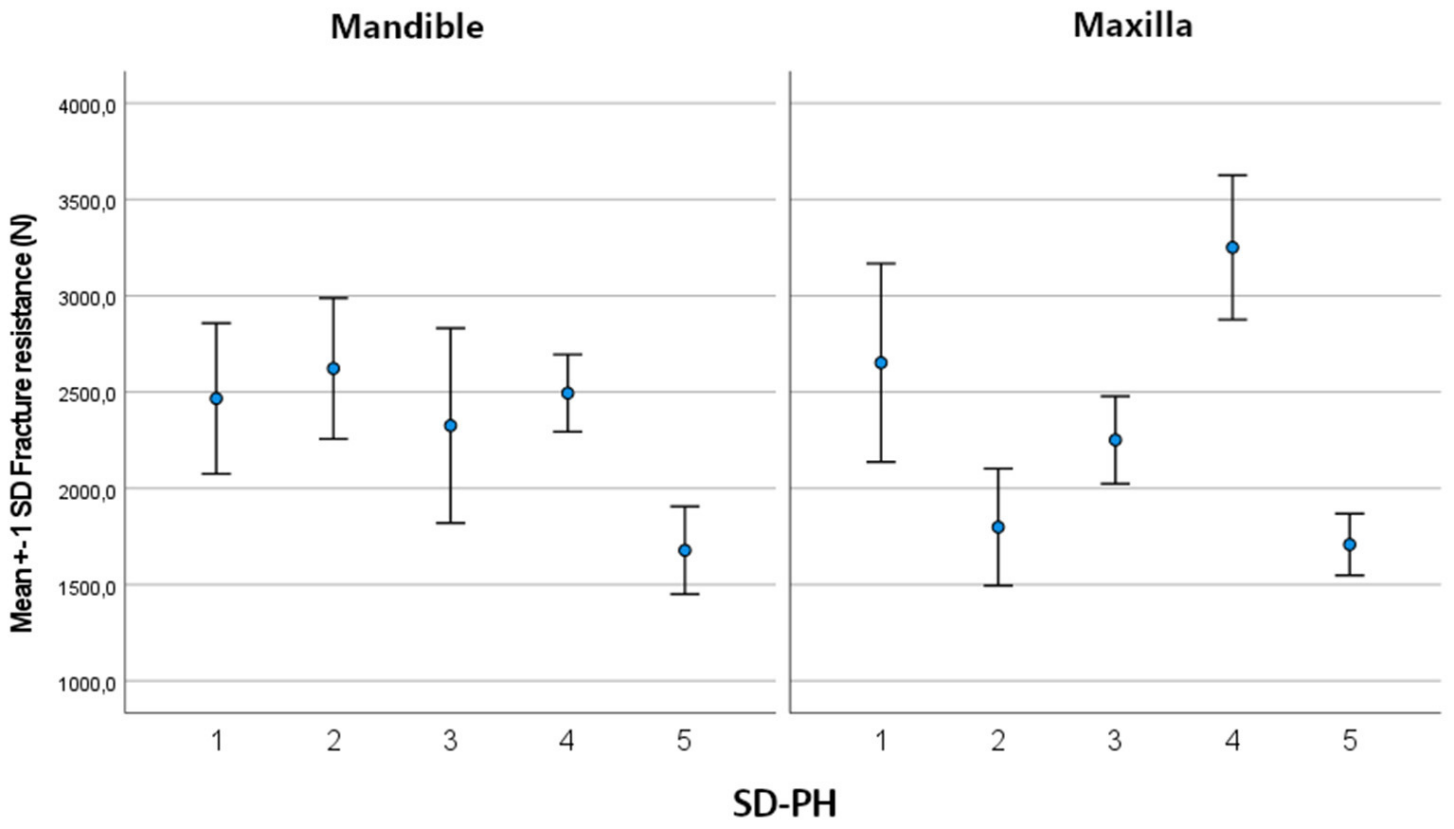
Bar chart illustrating the mean and SD of the fracture resistance in mandibular and maxillary cheek teeth at different locations on the tooth.

The mean maximum force sustained by the tested teeth was 2,373.34 ± 583.94 N (min: 1,422.7 N; max: 3,769.7 N). There was no significant difference in mean maximum force between mandibular (2,336.58 ± 485.59 N) and maxillary (2,410.73 ± 673.57 N) cheek teeth (*p* = 0.64). For both the mandible (*p* ≤ 0.01) and the maxilla (*p* ≤ 0.001), there was a significant variability between horses. In the mandible, the side of the tooth (buccal/ lingual) (*p* = 0.01) and location on the tooth (SD-PH 1-5) (*p* < 0.001) were significant factors in the univariate model (Supplementary Information 2). In the final multiple regression model of the mandible, the location on the tooth (SD-PH 1-5) (*p* < 0.001) and the Triadan number (*p* = 0.009) remained significant (Table 2). The highest fracture resistance was found on the level of the SD-PH 2, followed by SD-PH 4, 1, 3, and 5 (Figure 4). In the maxilla, the sustained maximum force was significantly different between locations on the tooth in the univariate model (*p* < 0.001) (Supplementary Information 2), which remained significant in the final multiple regression model (*p* < 0.001) (Table 2). In maxillary cheek teeth, the tooth had the highest fracture resistance at the level of the SD-PH 4, followed by SD-PH 1, 3, 2, and 5 (Figure 4). Additionally, a significant effect of the mesio-distal length of the occlucal surface (*p* = 0.01) on fracture resistance was withheld in the final multiple regression model.

**Table 2:** Results of the final multivariable model of factors significantly influencing the fracture resistance of cheek teeth in the first study population.

	**Category**	**Estimate**	**SE**	**95% CI**	***p*** **-value**
**Mandible**					
Intercept		2,428.06	148.80	2,136.43; 2,719.69	
SD-PH	1	Reference category			
	2	107.46	114.98	−117.89; 332.82	0.36
	3	−194.14	118.78	−426.94; 38.66	0.11
	4	53.70	155.80	−251.66; 359.05	0.73
	5	−756.40	−756.40	−995.28;−517.52	<0.001
Triadan	07	Reference category			
	08	110.68	106.14	−97.36; 318.72	0.30
	09	−196.34	124.64	−440.64; 47.96	0.12
	10	227.67	121.20	−9.87; 465.21	0.07
**Maxilla**					
Intercept		250.70	932.92	−1,577.78; 2,079.19	
SD-PH	1	Reference category			
	2	−687.95	159.85	−1,001.24; −374.65	<0.001
	3	−385.80	173.64	−726.13; −45.47	0.01
	4	501.97	138.73	230.07; 773.88	<0.001
	5	−737.35	143.87	−1,019.34; −455.37	0.01
Length	Cont.	82.68	32.93	18.13; 147.23	0.02

Observed fracture patterns are illustrated in Supplementary Information 3. The tooth broke above (*n* = 33/89; 37.1%), equal to (*n* = 29/89; 32.6%) or below (*n* = 27/89; 30.3%) the simulated bone level at similar frequencies. Significantly more fractures below the simulated bone level were recorded in maxillary (*n* = 19/44) compared to mandibular teeth (*n* = 8/45) (*p* = 0.001). No difference was observed between fracture levels in relation to different tested occlusal sites (SD-PH) on mandibular teeth. In maxillary teeth there was a significant difference in the number of fractures above and below the simulated bone level in relation to occlusal test sites (*p* = 0.02)(Supplementary Information 4).

**Figure 5:**
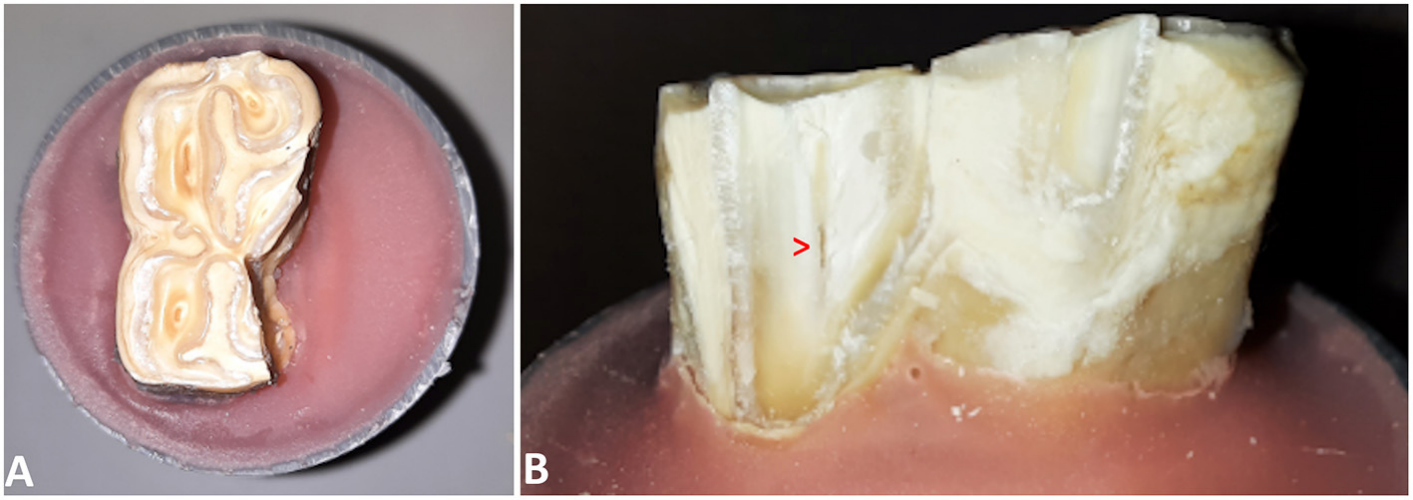
**(A)** Occlusal view of a 407 after fracture testing at the level of the secondary dentine associated with pulp horn 3. **(B)** Lingual view of the tooth demonstrating the exposed pulp cavity.

**Figure 6:**
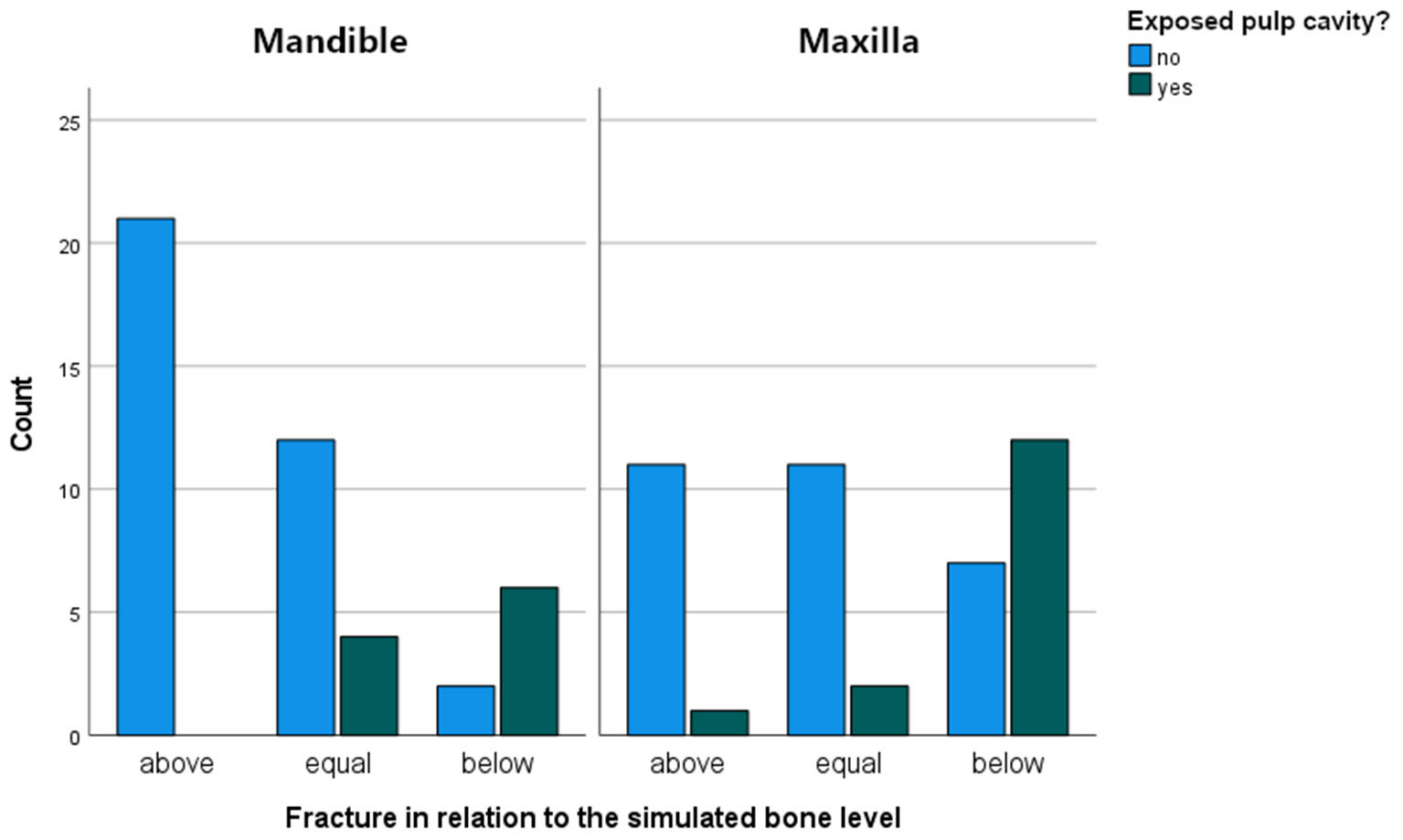
Bar chart illustrating the number of cases with exposed pulp cavities in relationship to the fracture level in the mandibular and maxillary cheek.

In 25/89 (28.1 %) cases the pulp cavity became exposed. In most cases, the pulp horn associated with the testing site was involved (20/25). Additionally, in 3 out of 20 cases, a second pulp horn was exposed. In 5/25 cases, a pulp horn became exposed which was not situated directly beneath the site where the pressure was exerted. It was observed that the location of pulp horn exposure was usually not at the most apical aspect of the fracture, but along the fracture plane (Figure 5). The mean age of teeth with an exposed pulp cavity was 8.64 ± 2.34 years old which was similar to the mean age of teeth without exposed pulp cavities (9.05 ± 1.84 years old, *p* = 0.5). Pulp cavities were exposed significantly more when the fracture level was below (*p* < 0.001) or equal to (*p* = 0.03) the simulated bone level. In only 1 out of 33 cases, a pulp horn was exposed after fracturing above the simulated bone level (Triadan 110, pulp horn 5, dental age 7 years). In 6 (out of 29) and 18 (out of 27) cases, the pulp cavity was exposed when the fracture was equal to and below the simulated bone level, respectively. The distribution of cases with exposed pulp cavities in relation to the fracture level is illustrated in Figure 6.

### Does the Presence of a Fissure Influence Fracture Resistance of Equine Cheek Teeth?

Teeth from 17 different horses were included in this part of the study. Forty-two cheek teeth (21 maxillary, 21 mandibular) with fissures were randomly selected. Mandibular cheek teeth had a mean dental age of 10.2 ± 1.6 years old (min 7 y, max 12 y) including 3 Triadan 07s, 4 Triadan 08s, 9 Triadan 09s and 5 Triadan 10s. Fissure classification involved type 1a in 12, type 1b in 8 and type 2 in 1 tooth, respectively. These teeth had an average occlusal surface width of 19.1 ± 1.9 mm (min 16 mm, max 22 mm) and an average occlusal surface length of 27.0 ± 1.7 mm (min 24 mm, max 30 mm). Maxillary cheek teeth had a mean dental age of 10.3 ± 1.4 years old (min 8 y, max 12 y) including 3 Triadan 07s, 4 Triadan 08s, 8 Triadan 09s and 6 Triadan 10s. Fissure classification involved type 1a in 14, type 1b in 5 and type 2 in 2 teeth, respectively. Their average occlusal surface width was 28.1 ± 1.9 mm (min 25 mm, max 31 mm) and their average occlusal surface length was 26.4 ± 1.9 mm (min 23 mm, max 31 mm). The average fissure depth of fissures in this population was 9.99 ± 5.43 mm (min 0.35; max 21.00 mm) and 8.83 ± 4.90 mm (min 2.73; max 20.60 mm) in mandibular and maxillary cheek teeth, respectively. A detailed overview of the distribution of teeth with fissures can be found in Supplementary Information 5. An age- and size matched tooth without fissures was selected as control (mean age of 10.00 ± 1.41 years and 10.14 ± 1.24 years; mean width of 19.0 ± 1.8 mm and 28.1 ± 1.5 mm; mean length of 27.1 ± 1.7 mm and 26.5 ± 1.8 mm of mandibular and maxillary control teeth, respectively).

**Figure 7:**
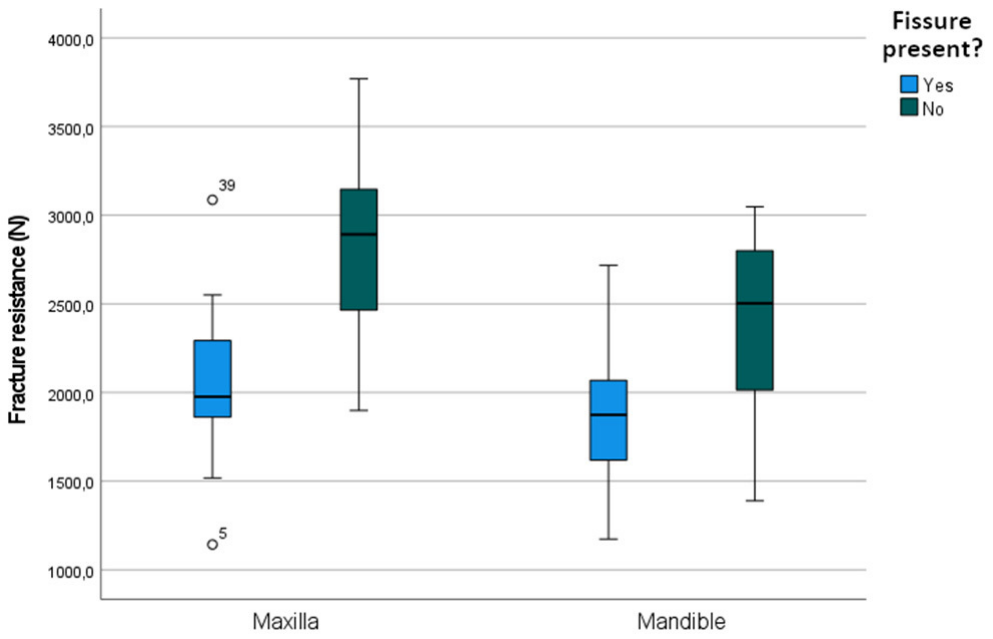
Boxplot illustrating the fracture resistance of cheek teeth with and without fissures present in the maxilla and mandible.

The mean maximum force sustained by teeth with fissures was 1,974.02 ± 402.09 N (min 1,142.9, max 3,087.0) compared to 2,594.70 ± 548.51 N (min 1,389.9, max 3,769.7 N) in teeth without fissures. The sustained maximum force was on average 529.27 N lower in cheek teeth with fissures (95% CI: 318.39; 740.16, *p* < 0.001). In the mandible, only fissure type was a significant predictor in the univariate analysis (*p* = 0.006). In the maxilla, the general presence of a fissure (*p* < 0.001) and fissure type (*p* < 0.001) were significant (Supplementary Information 6). In the final multiple regression model, fissure type remained significant in both the mandible (*p* = 0.006) and the maxilla (*p* < 0.001) (Table 3). The mean sustained maximum force in cheek teeth with and without fissures in the mandible and maxilla can be found in Figure 7. Furthermore, in mandibular teeth, location on the tooth (SD-PH 1-5) (*p* = 0.005), the age of the tooth (*p* = 0.006), the length of the tooth (*p* < 0.001) and the side of the jaw (left/ right) (*p* = 0.05) were significantly associated with a lower/higher fracture resistance. In maxillary teeth, only the location on the tooth (SD-PH 1-5) (*p* < 0.001) demonstrated a significant association, besides the earlier mentioned fracture type. Detailed results of the examined predictors can be found in Table 3.

**Table 3:** Final multivariable model of significant predictors on fracture resistance of the second study population.

**Variable**	**Category**	**Estimate**	**SE**	**95% CI**	***p*** **-value**
**Mandible**					
Intercept		−2,697.49	1,004.63	−4,666.52, −728.46	
Fissure	No	Reference category			
	Type 1a	–500.67	100.25	–697.16; –304.18	<0.001
	Type 1b	–625.74	116.65	–854.38; –397.11	<0.001
	Type 2	−121.07	277.34	−664.64; 422.51	0.67
SD-PH	1	Reference category			
	2	248.26	117.79	17.41; 479.12	0.04
	3	44.62	210.56	−368.06; 457.30	0.21
	5	–666.67	206.75	–1,071.90; –261.44	0.003
Age	Cont.	171.95	34.18	104.96; 238.94	<0.001
Length	Cont.	125.01	30.69	64.86; 185.16	<0.001
Jaw	Left	Reference category			
	Right	−161.06	90.32	−338.08; 15.96	0.08
**Maxilla**					
Intercept		2,231.03	211.29	1,816.91; 2,645.15	
Fissure	No	Reference category			
	Type 1a	–758.28	128.43	–1,010.00; –506.55	<0.001
	Type 1b	–536.78	209.19	–946.78; –126.79	0.01
	Type 2	–1,307.72	366.22	2,025.49; –589.95	<0.001
SD-PH	1	Reference category			
	3	185.10	240.86	−286.98; 657.17	0.45
	4	819.67	227.71	373.36; 1,265.98	0.001
	5	136.05	260.16	−373.85; 645.95	0.60

**Figure 8:**
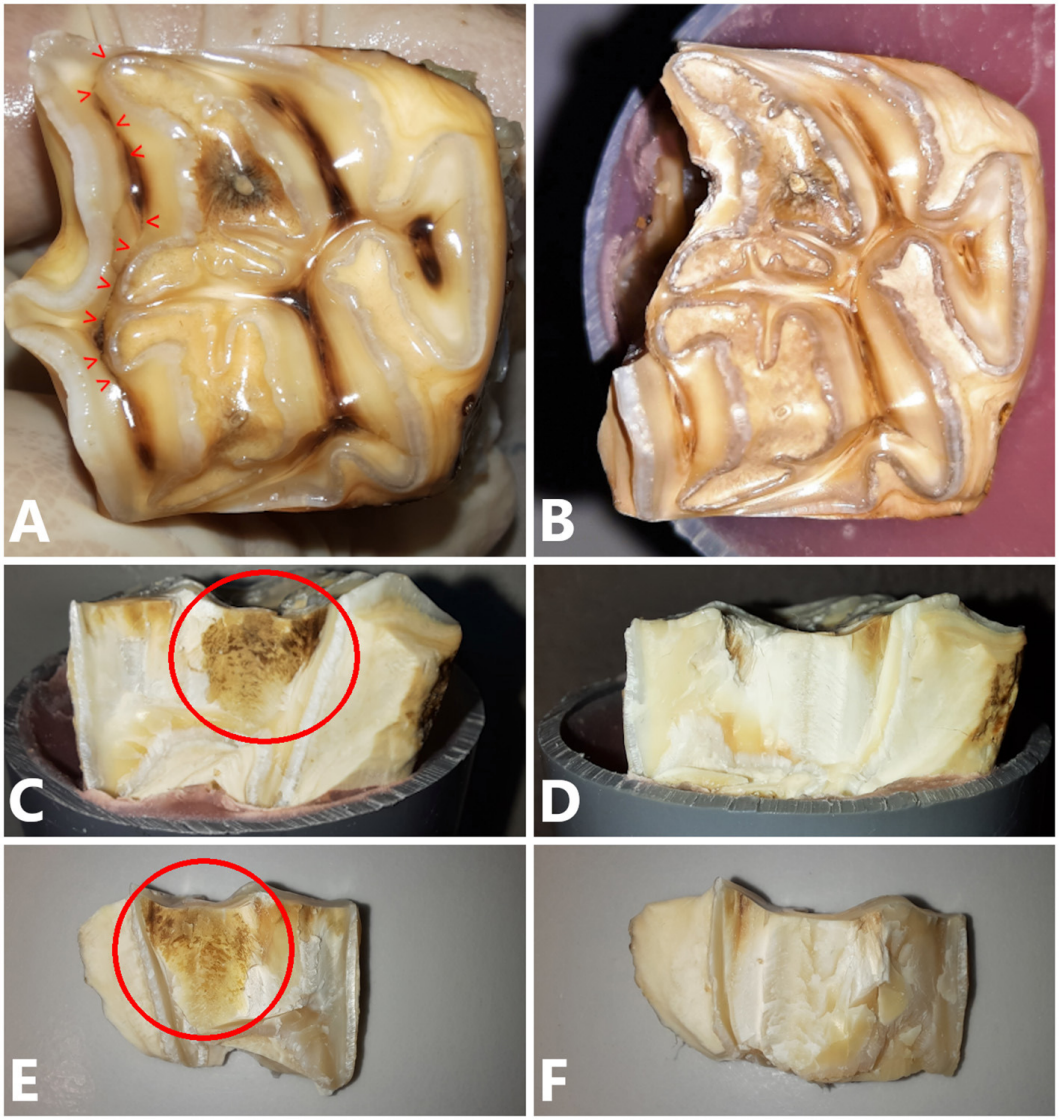
**(A)** Occlusal view of a 109 cheek tooth with a type 1b fissure present (red arrow heads). Pressure was exerted at the level of SD-PH 1. **(B)** Occlusal view of the tooth after fracture testing. The fracture followed the path of the fissure approximating SD-PH 2. **(C)** Lateral view of the tooth after fracture testing. A brown discolored area is visible (red circle) as well as on the fracture fragment **(E)**. **(D,F)** No discoloration was visible on the tooth and fragment of the matched control tooth.

The way the tooth fractured (fracture pattern) was grossly similar between teeth with and without a fissure in 14/21 paired maxillary cheek teeth and in 13/21 paired mandibular teeth. It was observed that the fracture plane of teeth with fissures was always discolored compared to teeth without fissures (Figure 8). There was no significant difference between fracture levels in cheek teeth with and without fissures (*p* = 0.25, *p* = 0.86 and *p* = 0.18 for the comparison of fracture levels above vs. below, below vs. equal and above vs. equal the simulated bone level, respectively). In 7 teeth, the pulp cavity became exposed (1 tooth with a fissure and 6 without).

## Discussion

This study examined the *ex vivo* fracture resistance of equine cheek teeth where individual (gender, age) and anatomical factors (Triadan number, the site of impact (SD-PH 1-5), dimensions of the occlusal surface) of the tooth were taken into consideration. A significant influence of the anatomical site where the impact on the tooth occurs in both maxillary and mandibular teeth was demonstrated, as was the Triadan number in mandibular teeth and the mesio-distal length of the occlusal surface in maxillary cheek teeth. Additionally, it was demonstrated that fissures on the occlusal surface decreased the fracture resistance in both mandibular and maxillary cheek teeth.

To the author's knowledge, this is the first study examining the *ex vivo* fracture resistance of equine teeth. The methodology used in this study is similar to most human-fracture resistance studies where the tooth is submitted to a continuously increasing force until it fractures. This type of impact is force regulated and carried out with a servo-hydraulic test machine (17). As this is an *ex vivo* study, there are of course limitations. The impact speed in the present study was set at 1 mm/min which is much lower than generated during the normal masticatory cycle. The test machine only allowed exerting forces in one direction which is in contrast to *in vivo* masticatory forces that act in 2 phases (vertical and oblique) during the chewing cycle (2, 3). Also, the set-up lacks the shock-absorbing properties of the periodontium (18, 19). Nevertheless, the fracture patterns generated in this experiment are highly similar to those described in previous studies (5, 12). This illustrates the clinical relevance of the model and also shows that it might be used for comparable experiments focusing on potentially weakening effects of other pathological factors (e.g., peripheral caries, infundibular caries, exposed pulp cavities) and provide an evidence-based approach for their treatment (e.g., infundibular restoration, root canal therapy). Another interesting future application involves research about dental floating. In equids, dental floating has become an established “routine” custom because of the perceived importance of the intervention by veterinarians and owners. Consequently, a critical evaluation of what is being achieved by so-called “occlusal equilibration” is often not performed (20) and evidence-based information on how that impacts the tooth's strength is not available. In human teeth, studies have emphasized the importance of maintaining dental structure to preserve the strength of the tooth (21, 22). One equine maxillary cheek tooth with excessive floating marks on the buccal side showed a fracture resistance of 1,093 N (at the level of the SD-PH 2) (unpublished data), which was lower than the tested cheek teeth in this study (min: 1,480 N on the same location). It therefore might be possible that the excessive floating had a negative impact on the fracture resistance of this tooth, which merits further investigation.

### *Ex vivo* Fracture Resistance of Equine Cheek Teeth With No Abnormalities

It has been suggested that the distribution of the mineralized tissues of an equine cheek tooth are a physiological requirement to cope with masticatory forces (23–25). An important finding in this study was the wide range in fracture resistance, with marked differences, depending on the region of the occlusal surface that was loaded. This indicates that there are anatomical areas with higher or lower intrinsic structural strength on the occlusal surface of equine cheek teeth. Kilic et al. reported that the enamel thickness was larger at the buccal aspect of the maxillary and the lingual aspect of the mandibular cheek teeth (23). However, Windley et al. recorded the largest thickness of enamel on the buccal side of mandibular teeth (26). Clinical papers reporting prevalence of cheek tooth fractures reflect these contradictory structural results. Uncomplicated crown fractures of both mandibular and maxillary cheek teeth were more frequently observed on the lingual side in one study (12), whereas idiopathic buccal slab fractures were more frequently reported in others (6, 8). These inconsistent findings illustrate the complex multifactorial mechanism behind development of a tooth fracture. Our results indicate that a mandibular cheek tooth was the strongest on the buccal side of the tooth at the level of the SD-PH 2 (mean sustained maximum force of 2,622.682 N), followed by SD-PH 4 (2,494.74 N), SD-PH 1 (2,466.76 N), SD-PH 3 (2,326.15 N) and SD-PH 5 (1,678.35 N). In maxillary cheek teeth, the tooth was the strongest at the level of the SD-PH 4 (mean sustained maximum force of 3,251.06N), followed by SD-PH 1 (2,652.48 N), SD-PH 3 (2,251.52 N), SD-PH 2 (1,798.83 N) and SD-PH 5 (1,708.42 N). When comparing with the prevalence of uncomplicated fracture locations these results can explain why these fractures were more observed on the lingual side (12).The lower recorded fracture resistance at SD-PH2 level in maxillary cheek teeth also coincides with the higher prevalence of partial buccal slab fractures at this location compared to SD-PH1 (12). Kilic et al. furthermore reported that the overall enamel thickness in maxillary cheek teeth is thicker compared to mandibular cheek teeth which could suggest that maxillary cheek teeth are more capable of coping with masticatory forces (23). However, this was not supported in this study since the average fracture resistance of mandibular and maxillary cheek teeth was comparable (2,336.58 and 2,410.73 N, respectively). This might suggest that, in equine cheek teeth, the enamel thickness is not the primary factor determining fracture resistance.

The forces generated during equine mastication are reported to reach 1,956 N in young horses (3). The average *ex-vivo* fracture resistance in this study was higher (2,373.34 N), indicating that healthy teeth are overall well-equipped to cope with normal masticatory forces. Masticatory forces have been reported to increase from rostral to caudal (3), therefore it could be conjectured that teeth situated more caudally in the mouth should have a higher resistance to fracture. In the mandible, the Triadan number was a significant factor influencing the fracture resistance, with Triadan 10 (second molar) indeed displaying the highest ability to withstand fracture. However, Triadan 09 (first molar) had the lowest fracture resistance which does not follow this hypothesis. This tooth number has been reported as the one most frequently fractured (27), but this was not consistent with other studies (6, 8, 9). In the maxilla, the mesiodistal length of the occlusal surface was also a significant factor, with an increasing fracture resistance with increasing length. This demonstrates that larger teeth can be expected to sustain higher masticatory forces without fracture. It is somewhat surprising that factors that significantly influence the fracture resistance differ between the mandible and the maxilla, especially since both the triadan number and the mesiodistal length represent the amount of tooth that is present in and around the region of the testing device (28). This might be explained due to the different anatomy and distribution of dental tissues between the more narrow mandibular and wider maxillary cheek teeth. Ultrastructural differences between mandibular and maxillary teeth (e.g., the presence of a different ratio of enamel types) might further explain these differences (23). It has been demonstrated that the mesiodistal occlusal distance varies by triadan number (28), however these differences are relatively small and other ultrastructural characteristics between triadan numbers might be of more importance in regard to the tooth's capacity to withstand loading forces. Ultrastructural studies comparing the toughness of dental tissues in between tooth positions are unfortunately lacking to provide further insights.

Tooth age appeared to have no influence on the fracture resistance of cheek teeth in this study population. However, it has to be noted that the age-range of this study population (5–12 years) might not be wide enough to detect significant differences. In this age-range a large variety of pulp configurations (most commonly a separation into mesial and distal pulp compartments) is possible and therefore also a large variety of the volume of dentin (29). It might be possible that, for example very young teeth (< 5 years, with a common pulp chamber) do have a different fracture resistance. It would therefore be interesting to examine the effect of the pulpar anatomy of the tooth (and the volume of dentin) on its fracture resistance.

### Does the Presence of a Fissure Influence Fracture Resistance of Equine Cheek Teeth?

The presence of occlusal fissures significantly decreased the ability of cheek teeth to withstand loading forces. This was especially seen for type 1a and 1b fissure in mandibular teeth, and for all fissure types in maxillary teeth. These results support the findings of a longitudinal *in vivo* study, where cheek teeth with fissures were observed to have higher odds to fracture (12). There is a big variation in the occluso-apical depth of fissures, but this did not appear to influence fracture resistance. The observed brown discoloration of fissure fracture walls was demonstrated to be caused by plant material in a histological study (30).

Inconsistent results were recorded for the loading experiments on healthy teeth from the first and second group (Table 2 vs. Table 3). These differences are most-likely attributed to the difference in positioning of the tip of the loading device between groups. This shows that standardization of the experimental set-up is extremely important for future research in order to produce reliable test results. The finding that the mesiodistal length was significant in the mandibular teeth in this study population, but not triadan number (as was the case for the non-fissure model) could also suggest some form of interrelationship between these factors. However, in none of the models where the combined effects of several predictors on fracture resistance were evaluated, these predictors were both significant. Other contributing factors might include spatial variation in dentin and enamel thickness which could influence the fracture resistance in areas relatively close to each other.

### Fracture Level and Exposed Pulp Cavities

Of clinical interest was the significant higher observation of exposed pulp horns when the fracture occurred below and equal to the simulated bone level. This is in agreement with the reported location of the occlusal aspect of the pulp horn, that often lies just beneath the gingival margin (31). These findings suggest that when a fractured equine cheek tooth is diagnosed which involves the intra-alveolar part of the tooth, a thorough examination of the fracture plane is important to verify pulp horn exposure. This inspection should not only be done visually, but also includes probing the entire fracture plane with a sharp instrument (dental explorer/Hedstrom file) since the communication with the pulp cavity can be very small. It is the authors' personal experience that in more chronic cases, careful removal of superficial plaque from the surface is sometimes required before being able to identify pulp exposure. The same procedure accounts for any level of fracture plane as a wide variation in subocclusal secondary dentine thickness has been reported (31–33). In the present study, an exposed pulp horn related to a fracture above the simulated bone level was observed in one case. Age did not have a significant effect on whether a pulp horn became exposed or not in this study population, which is supported by the absence of age-related changes in subocclusal secondary dentine thickness (31, 32). Finally, the presence of an occlusal fissure did not influence the resultant fracture level. However, it was noteworthy that only 1 (out of 42) tooth with a fissure was observed to have an exposed pulp (in contrast to 6/42 teeth without fissures in the matched control group). This might suggest that a fissure-to-fracture evolution is less likely to result in an exposed pulp horn in contrast to a tooth that fractures without the previous presence of a fissure. This might be related to stimulation of the pulp to produce tertiary dentin in the presence of an adjacent fissure. However, due to the relative low number of these observations, it is not possible to draw definite conclusions on this matter.

## Conclusion

The methodology used in this study provides an *ex vivo* experimental set-up to test fracture resistance of equine cheek teeth which can be used for future research to examine the potentially weakening effect of dental pathology and to provide an evidence-based approach for their treatment. This study showed that there are anatomical sites of weakness on the tooth. Additionally, it was demonstrated that fissures on the occlusal surface decreased fracture resistance, independently of their depth.

## Data Availability Statement

The raw data supporting the conclusions of this article will be made available by the authors, without undue reservation.

## Ethics Statement

Ethical review and approval was not required for the animal study because The study was performed on material obtained from cadavers of deceased animals (unrelated to this study) and from an abattoir.

## Author Contributions

EP, LV, SR, and RC designed the study. EP performed the execution of the study (collecting teeth, preparing teeth, teeth testing and processing data). BB analyzed the data. EP, LV, and BB interpreted the findings. EP and LV prepared the manuscript. All authors contributed to the article and approved the submitted version.

## Funding

The Special Research Fund of Ghent University (BOF-UGent) is acknowledged for the financial support for the Centres of Expertise UGCT (BOF.EXP.2017.0007). This research was further funded by the Department of Surgery and Anaesthesiology of Domestic Animals, Faculty of Veterinary Medicine, Ghent University.

## Conflict of Interest

The authors declare that the research was conducted in the absence of any commercial or financial relationships that could be construed as a potential conflict of interest.

## Publisher's Note

All claims expressed in this article are solely those of the authors and do not necessarily represent those of their affiliated organizations, or those of the publisher, the editors and the reviewers. Any product that may be evaluated in this article, or claim that may be made by its manufacturer, is not guaranteed or endorsed by the publisher.

## Abbreviations

SD-PH: secondary dentine above pulp horn.
