# Supplementary material for: Fracture Resistance of Equine Cheek Teeth With and Without Occlusal Fissures: A Standardized ex vivo Model
Source: Front Vet Sci. 2021 Sep 7;8:699940. doi: 10.3389/fvets.2021.699940 (PMC8453076; doi:10.3389/fvets.2021.699940)
Supplement: Supplementary file 1 [file Table_1.PDF]

Supplementary Information 1. An overview of teeth included in the study to examine factors that might influence the fracture resistance of equine cheek teeth.

Mandibular cheek teeth

Horse	Triadan	Tooth age (years)	Site(s) of exerted pressure (SD-PH)
6	409	9	1
2	409	11	1
7	410	11	2
7	310	11	1, 5
2	309	11	1
1	409	12	5
6	407	7	2, 3
5	309	12	3
6	410	8	5
4	407	6	3
4	308	5	2, 3
4	408	5	5, 1
4	410	7	4, 1
6	310	8	4
6	308	6	4, 2
4	409	8	1, 5
7	307	10	1, 5
2	307	9	2, 3
2	308	8	2, 3
2	408	8	1, 5
5	307	10	4
5	407	10	2, 3
5	308	9	4, 1
1	407	10	1, 2
7	407	10	2, 3
7	308	9	5, 3
2	310	10	3, 2
7	409	12	2

Maxillary cheek teeth

Horse	Triadan	Tooth age (years)	Site(s) of exerted pressure (SD-PH)
3	110	12	3
2	107	9	4
4	108	5	4
5	207	10	4
2	109	11	4
2	210	10	4, 1
2	209	11	4, 1
2	108	8	4, 1
1	208	9	1
7	210	11	1
5	209	12	1
4	210	7	1, 5
7	108	9	1
5	109	12	2
2	207	9	1
5	208	9	1
2	110	10	4, 1
4	110	7	2, 5
6	107	7	1, 5
6	208	6	3
6	108	6	3
6	110	8	3
7	209	12	1, 5
4	208	5	3, 2
1	110	11	4
7	110	11	2, 5
5	107	10	1, 3
5	108	9	2, 5
7	107	10	5
7	207	10	5
7	208	9	5, 2
