# Supplementary material for: Fracture Resistance of Equine Cheek Teeth With and Without Occlusal Fissures: A Standardized ex vivo Model
Source: Front Vet Sci. 2021 Sep 7;8:699940. doi: 10.3389/fvets.2021.699940 (PMC8453076; doi:10.3389/fvets.2021.699940)
Supplement: Supplementary file 2 [file Table_2.PDF]

Supplementary Information 2. Corrected P-values of single predictors. Detailed results of significant factors are provided.

	Mandible	Maxilla
Gender	1.0	1.0
Left/right	1.0	1.0
Triadan	0.99	1.0
Age	1.0	1.0
Lingual/Buccal	**0.01**	1.0
SD-PH	**< 0.001**	**< 0.001**
Width	1.0	1.0
Length	1.0	0.79
Square	1.0	1.0

Mandible univariate model

	Category	Estimate	SE	95% CI	p-value
**Side tooth**	Buccal	Reference Category			
	**Lingual**	**-377.29**	**113.40**	**-599.54; -155.035**	**0.002**
**SD-PH**	1	Reference Category			
	2	160.58	122.56	1.31; 0.20	0.20
	3	-157.91	125.33	-1.26; 0.22	0.22
	4	207.56	163.93	1.27; 0.21	0.213
	**5**	**-715.03**	**133.94**	**35.79; -5.34**	**< 0.001**

Maxilla univariate model

	Category	Estimate	SE	95% CI	p-value
**SD-PH**	1	Reference Category			
	**2**	**-733.30**	**170.19**	**-1066.86; -399.74**	**< 0.001**
	3	-318.04	182.11	-674.97; 38.88	0.09
	**4**	**476.47**	**147.93**	**186.54; 766.40**	**0.003**
	**5**	**-764.49**	**153.53**	**-1065.37; -463.56**	**< 0.001**
