# Supplementary material for: Fracture Resistance of Equine Cheek Teeth With and Without Occlusal Fissures: A Standardized ex vivo Model
Source: Front Vet Sci. 2021 Sep 7;8:699940. doi: 10.3389/fvets.2021.699940 (PMC8453076; doi:10.3389/fvets.2021.699940)

Supplementary Information 3. Fracture patterns observed after *ex vivo* fracture testing (the location of the testing site is illustrated by the red dot on the left tooth). Green and yellow lines represent uncomplicated fracture patterns. Red and purple lines represent complicated fracture patterns.

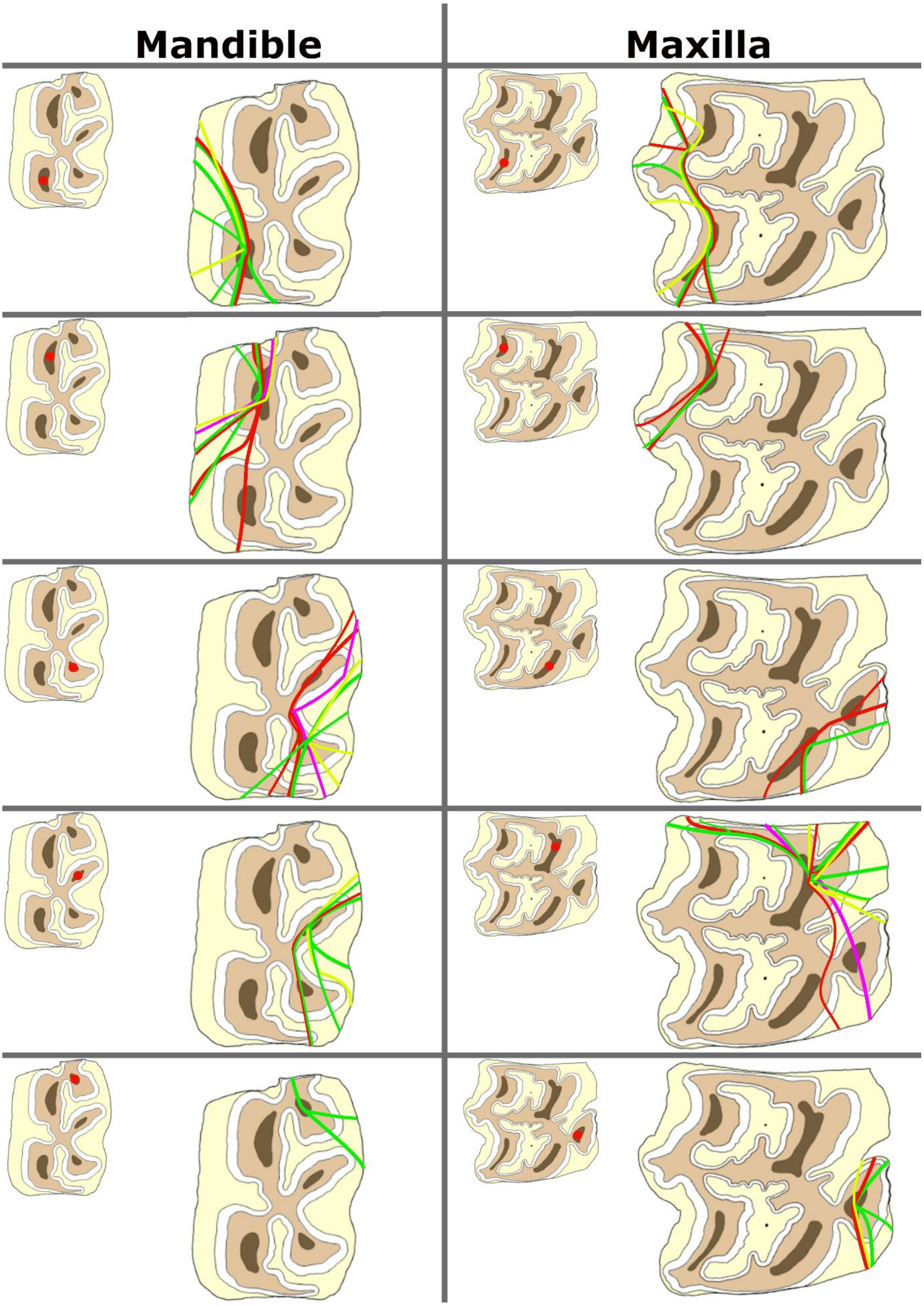

Supplement: Supplementary file 3 [file Table_3.PDF]
