# Supplementary material for: Fracture Resistance of Equine Cheek Teeth With and Without Occlusal Fissures: A Standardized ex vivo Model
Source: Front Vet Sci. 2021 Sep 7;8:699940. doi: 10.3389/fvets.2021.699940 (PMC8453076; doi:10.3389/fvets.2021.699940)
Supplement: Supplementary file 4 [file Table_4.PDF]

Supplementary Information 4.

Number of fractures above, below or equal to the simulated bone level.

	SD-PH	Above	Below	Equal	Total
**Mandible**					
	**1**	6	1	4	11
	**2**	2	4	5	11
	**3**	5	1	4	10
	**4**	3	1	1	5
	**5**	5	1	2	8
**Maxilla**					
	**1**	2	9	3	14
	**2**	1	2	3	6
	**3**	0	4	2	6
	**4**	3	3	3	9
	**5**	6	1	2	9

Results of the mixed model of fracture level versus tested location on the tooth (SD-PH)

		Estimate	Std. Error	p-value
**Mandible**				
**Above vs below**				0.24
**Below vs Equal**				0.89
**Above vs Equal**				0.58
**Maxilla**				
**Above vs below**				**0.019**
	**1 (intercept)**			
	**2**	8.109e-01	1.453e+00	0.58
	**3**	-3.180e+01	8.524e+06	1.00
	**4**	1.504e+00	1.130e+00	0.18
	**5**	3.296e+00	1.333e+00	**0.01**
**Below vs Equal**				0.89
**Above vs Equal**				0.58
