# Supplementary material for: Fracture Resistance of Equine Cheek Teeth With and Without Occlusal Fissures: A Standardized ex vivo Model
Source: Front Vet Sci. 2021 Sep 7;8:699940. doi: 10.3389/fvets.2021.699940 (PMC8453076; doi:10.3389/fvets.2021.699940)

Supplementary Information 5. Overview of teeth with fissure fractures present.

Mandibular cheek teeth

Horse	Triadan	Tooth age (years)	Fissure type	SD-PH	Fissure depth (mm)
6	309	9	1a	1	17.95
10	408	9	1b	2	10.26
11	409	10	1a	1	21.00
1	409	12	1a	1	19.11
15	310	11	1a	2	13.49
13	408	9	1a	2	5.39
11	307	8	1b	2	3.72
11	408	7	1b	2	10.94
10	407	10	1b	2	9.75
7	309	12	1a	1	7.38
5	310	11	1b	3	5.06
1	309	12	1a	1	12.23
5	309	12	1a	1	9.77
6	410	8	1a	1	7.23
7	408	9	1b	2	6.75
15	409	12	1b	2	18.28
13	310	11	1a	1	8.17
13	309	12	1a	1	7.74
8	307	9	2	NA	0.35
16	410	10	1a	1	5.04
15	309	12	1b	5	10.24

Maxillary cheek teeth

Horse	Triadan	Tooth age (years)	Fissure type	SD-PH	Fissure depth (mm)
10	109	12	1b	5	3.41
17	207	9	1a	4	2.97
15	209	12	2	NA	3.13
7	108	9	1a	4	9.82
5	208	9	2	NA	5.16
5	210	11	1b	3	4.29
5	110	11	1b	3	3.59
5	109	12	1a	4	9.91
14	107	10	1a	4	3.27
10	108	9	1a	4	14.14
10	208	9	1a	4	20.6
7	210	11	1a	4	13.46
3	110	12	1a	4	11.91
7	109	12	1a	4	12.51
16	209	11	1a	4	11.09
6	109	9	1b	1	9.18
6	209	9	1b	1	8.12
6	210	8	1a	4	12.65
5	209	12	1a	4	10.39
2	207	9	1a	4	13.06
15	110	11	1a	5	2.73

Graphical overview of involved SD-PH and fissure types. (NA = not applicable)

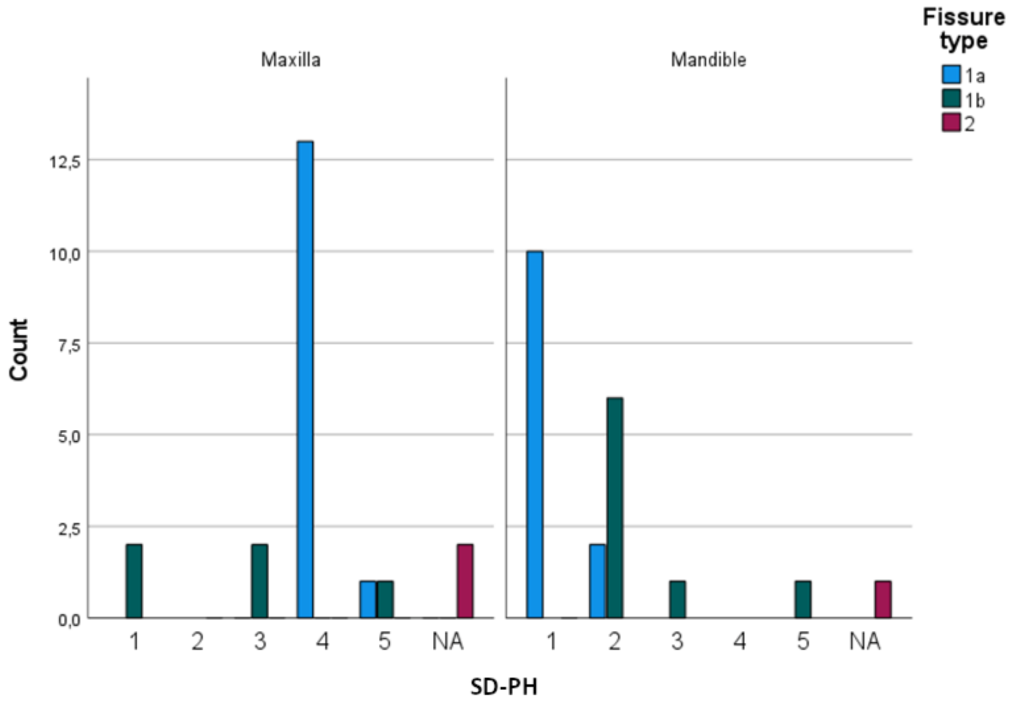

Supplement: Supplementary file 5 [file Table_5.PDF]
