# Supplementary material for: Fracture Resistance of Equine Cheek Teeth With and Without Occlusal Fissures: A Standardized ex vivo Model
Source: Front Vet Sci. 2021 Sep 7;8:699940. doi: 10.3389/fvets.2021.699940 (PMC8453076; doi:10.3389/fvets.2021.699940)
Supplement: Supplementary file 6 [file Table_6.PDF]

Supplementary Information 6. Corrected P-values of single predictors. Detailed results are provided of significant factors.

	Mandible	Maxilla
Fissure (yes/no)	0.08	**< 0.001**
Fissure type	**0.006**	**< 0.001**
Fissure depth	0.07	0.28
Gender	1.0	1.0
Left/right	1.0	1.0
Triadan	1.0	1.0
Age	1.0	1.0
Lingual/Buccal	0.97	0.93
SD-PH	0.40	0.10
Width	1.0	1.0
Length	0.09	1.0
Square	0.68	1.0

Mandible univariate model

	**Category**	**Estimate**	**SE**	**95% CI**	**p-value**
**Fissure type**	**None**	Reference category			
	**1a**	**-420.17**	**113.39**	**-698.72; -141.62**	**0.005**
	**1b**	**-620.40**	**16223**	**-938.36; -302.44**	**< 0.001**
	2	550.57	313.01	-62.92; 1164.07	0.09

Maxilla univariate model

	Category	Estimate	SE	95% CI	p-value
Fissure present	Yes	Reference category			
	**No**	**695.68**	**150.95**	**399.83; 991.54**	**< 0.001**
Fissure type	None	Reference catergory			
	**1a**	**-523.11**	**152.15**	**-821.32; -224.90**	**0.001**
	**1b**	**-997.77**	**231.83**	**-1452.14, -543.40**	**< 0.001**
	**2**	**-1284.12**	**311.97**	**-1895.57; -672.67**	**< 0.001**
