# Supplementary figures and images for: Fracture Resistance of Equine Cheek Teeth With and Without Occlusal Fissures: A Standardized ex vivo Model
Source: Front Vet Sci. 2021 Sep 7;8:699940. doi: 10.3389/fvets.2021.699940 (PMC8453076; doi:10.3389/fvets.2021.699940)

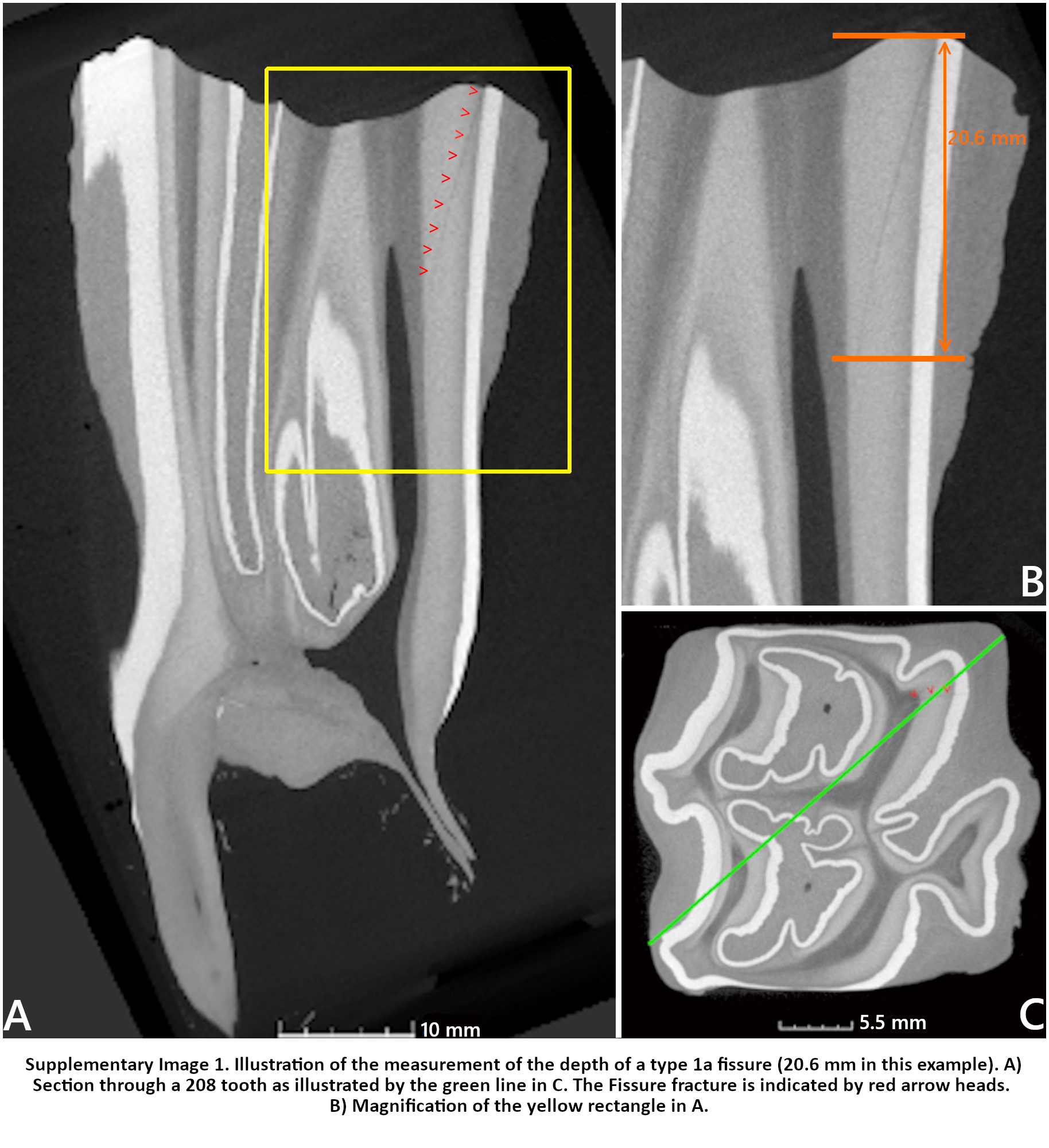

Supplement: Supplementary file 7 [file Image_1.TIFF]
